# The Future of Palliative Treatment in India: A Review

**DOI:** 10.7759/cureus.29502

**Published:** 2022-09-23

**Authors:** Adinath Gaikwad, Sourya Acharya

**Affiliations:** 1 Department of Medicine, Jawaharlal Nehru Medical College, Wardha, IND; 2 Department of Medicine, Datta Meghe Institute of Medical Sciences (Deemed to be University), Wardha, IND

**Keywords:** india and palliative care, structure of palliative care, future of india, palliative and supportive care, end of life and hospice care

## Abstract

The rapid dynamics of the world bring about changes in all aspects of the life of human beings. These dynamics hence call for the need for research and development in the field of the betterment of the life of a human. Palliative care for the patient in need is a field that drastically affects human life and can provide an ethical and more humanitarian way to live. The main objective of this article is to examine the Indian system of palliative care's organizational structure, roles, and applicability in India. By reading various research articles on the internet, mainly PubMed and Google Scholar, we collected enough information on the palliative care facilities in India. After going through the research articles and thorough discussion, it comes to the knowledge that India's existing palliative care facilities are lacking behind the rest of the world. However, some states have better approaches and facilities, but overall research suggests the need for more development in the field of palliative care even more. India needs a systematic approach to improving palliative care. These improvements include increasing the workforce, better application of policies, good political support, and educating health care workers and patients requiring palliative treatment.

## Introduction and background

What is palliative treatment?

Regardless of the age, diagnosis, or outlook, “Patients receiving specialized medical care for severe diseases were given relief from the symptoms, suffering, and consequences involved with their conditions” [1]. Children’s palliative care comprises providing healthcare activities for the child’s health, mind, and soul and supporting the family [2]. One of the primary parts of palliative care is setting appropriate goals for clinical scenarios with poor prognoses. “Life is worth living every day until the last one.”

Components of palliative treatment

**Figure 1:**
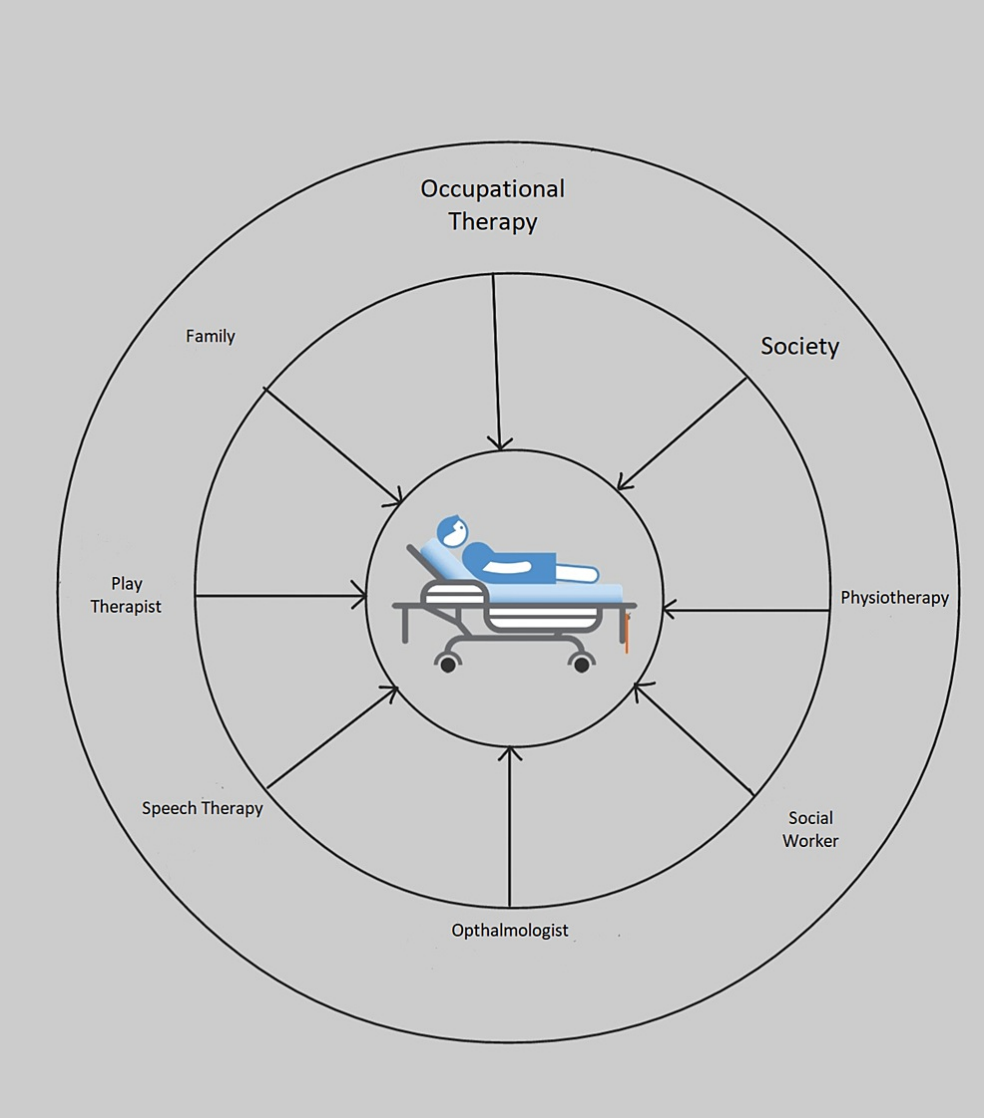
Spoke wheel model of palliative treatment Image credit: The authors of the current study.

Patients, as well as their loved ones and friends, are involved in palliative care. Some essential components of palliative treatment are mentioned in Figure 1. All personnel who provide medical care treatment are primarily responsible for ensuring that patients die with dignity and should get the support they need from family and friends. Effective palliative cancer treatment requires an integrated team that can provide treatment in all patient environments and in a cost-effective way. This can help provide quality care at medical centers, emergency and elective care institutions, and private residences [3].

Figure 1 shows a spoke wheel model for palliative care, which has patients at the center. Each spoke has components of palliative care such as physicians, chaplains, social workers, technicians, nurses, pharmacists, psychologists, therapists, and many more. All these components work together for the betterment of the patients.

Non-drug palliative care

There are supportive, cognitive, behavioral, and physical methods, which are shown in Table 1 [4]. These are some facilities provided to the patient, which do not involve any pharmaceutical intervention in palliative care. These interventions are as necessary as the facilities involving pharmaceutical interventions.

**Table 1:** Non-drug palliative care *Direct application of heat and cold may harm the children.

Supportive Methods	Cognitive Methods	Behavioral Methods	Physical Methods
Family-centered care	Distraction	Deep breathing	Touch
Information	Music	Relaxation	Heat and cold*
Empathy	Image therapy		Transcutaneous electrical nerve stimulation (TENS)
Play	Hypnosis therapy		

Who needs palliative treatment?

According to estimates, just 14% of individuals who require palliative care do so globally [5]. Each year, around seven million Indians with terminal conditions may need palliative care therapies to relieve the refractory symptoms that have not responded to prior treatments and are present toward the end of life [6]. Physical discomfort often signals a person’s most important worry. According to experts, millions of cancer patients have moderate to severe pain annually, as do tens of thousands of HIV/AIDS patients [7,8]. Other typical signs of these fatal illnesses include nausea, shortness of breath, anxiety, and depression. Demographically, the proportion of fatalities requiring palliative care increased from 72.5% in 2006 to 74.9% in 2014 [2]. Moreover, the need for palliative care will increase from 38.0% in 2014 to 56.0% by 2040. Annually, 40 million people require palliative care. Around 78% of persons needing palliative care reside in low- and middle-income economies [9,10]. DR-TB, hemorrhagic fevers, and injury are specialized populations that should also receive palliative care. Even though palliative care research is growing at an exponential rate, the implementation of this new knowledge on the ground level in India is at a much slower rate when compared to the rest of the world.

Why should one receive palliative care?

Communication problems, care coordination and planning, as well as psychological, emotional, and spiritual support are more or less concerned in this field [11]. Palliative care is helpful to get complete relief from physical (pain) and psychological symptoms, and it is crucial to provide palliative care and pain management measures safely and effectively. It includes usage of medications and other tools, which may be appropriately given or used in a primary care context [10].

Gains From Palliative Care

The contact between patients, caregivers, and medical personnel is improved through palliative medicine. Palliative medicine not only provides emotional and spiritual support but also creates a network of assistance to allow the sufferer to live as self-dependent as possible, i.e., it makes patients autonomous and active. Palliative care has increased the caretaking of patients and their loved ones with therapy. It improves medical care quality while cutting expenses. It allows people to maintain their comfort level by preventing and reducing pain and suffering. Palliative treatment facilitates communication between sufferers and their loved ones.

Gains From Early Palliative Care

Patients who are found to have incurable cancers in the early stage benefit from the application of palliative care as early as possible in terms of improving life, reducing depressive symptoms, improving prognosis tolerating, and compelling conversation about end-of-life care choices [12]. Early palliative treatment significantly enhanced the life and psychological state of patients with higher staged lung cancer. Patients who received early palliative care survived longer than patients who received conventional treatment but required less vigorous care in the last phases of their illness [13].

Disadvantages of Late or Inappropriate Palliative Care

A few disadvantages of late or inappropriate palliative care include families' misconceptions of palliative care, poor doctor-patient communication, and families' insufficient readiness to deteriorate patients' situations.

Context

According to the availability of palliative care, India scored 67th place out of 80 countries [14]. This ranking of the countries is done on aspects such as relevant regulations, enough medicine supply, training of healthcare personnel, public awareness campaigns, and all stages of implementation of palliative care services in the field of work [15]. Recognizing, finding, diagnosing, assessing, and treating pain and emotional stress; addressing end-of-life issues; and managing the suffering and distress of carers are also some factors that signify the level of palliative treatment provided in a region. Furthermore, the most crucial method in providing primary palliative care is creating a plan of action based on available resources [16,17].

## Review

Objectives

The main objective of this article is to examine the Indian system of palliative care's organizational structure, roles, and applicability in India. Rapid dynamics in the world order in almost every aspect bring about dynamics in the existing diseases and their treatment. Similar to the recent example of COVID-19, many diseases may erupt out of nowhere. Some disorders have therapy in the experimental stage, but illness at the end stage of the condition is untreatable. This is where the unmet need for palliative care arises. This review is one small step to gaining more knowledge about the facilities providing hospice, palliative care, and end-of-life care in India compared to the dynamics happening with the population, development, and research in the world. It includes the people in requirement of palliative care, available facilities that provide palliative care, the challenges in improving palliative care in India, the future of palliative care in India, and, lastly, some new ideas to offer better palliative treatment in India.

Palliative treatment in India

According to a recent survey, more than 108 entities currently provide facilities to improve the quality of life and palliative treatment services in 16 states/union territories [18]. These are mainly restricted to major cities and regional cancer centers, with the exception of Kerala, where services are more readily available than in other states. NGOs, public and private hospitals, and hospices are primary care providers. Services have developed unevenly, with the south having more services than the north. The majority of states have subpar coverage. Palliative care is hardly used in 19 states or union territories [18]. Health care facilities in Kerala can be the only example where every district is equipped with the instruments to provide palliative care. There are still some glaring deficiencies that involvement of the home-care services may decrease the involvement of the professional health workers. The unprofessional workforce can risk the treatment of the patient and also risk the research by non-documentation of the treatment [19].

Unlike Kerala, in Utter Pradesh, only 14 practitioners (28%) could specify more than three crucial aspects of end-of-life healthcare. Only two doctors (4%), none of whom had undergone training, used palliative care services. Most of the time, doctors' explanations and counseling fell short of the expectations of patients and their loved ones. They were only partially satisfactory; 95% of end-stage cancer patients wanted to use specialized services but did not know the existence of palliative care-providing institutes. There was no explicit health program in the Government of Uttar Pradesh's plan. In Lucknow, there was just one philanthropic organization for elderly cancer patients in need [20]. For geriatric people, tertiary care hospitals offer most outpatient department (OPD) services for the elderly. As urban areas are developed more than rural areas, most facilities like government institutions, including daycare centers, senior living communities, counseling centers, and recreational centers, are situated out of reach of the rural population. In a study to look at the needs and requirements of older people in rural Meerut, it was shown that 96% of participants had never used any geriatric welfare services and that up to 46.3% were unaware of any specialized services for the aging population nearby their place of residence. The nearest government building, about 59% of them, is 3 km away from their houses [21]. The inability of older people to access the available health services is caused by a lack of transportation options and their reliance on others to accompany them to the medical institution.

Algorithm for providing palliative care in India

While giving palliative care to patients, specific roles should be performed by the physician, integrated team, and family. The role of the physician is not only subjective and objective evaluation of medical personnel but also to get early, accurate, and truthful prognostic communication and early provision of palliative care when adverse results are anticipated [22]. The role of an integrated team is to achieve* *open, early, and repeated conversations leading to consensus. Transparency and accountability are ensured and expected within the caregiving team through proper recordkeeping [22]. The role of the patient/family is to* *decide and conflict resolution through good communication; after consulting with the family, life support is withheld or removed. Education about end-of-life symptomatology and end-of-life care procedures is also crucial for the family members [22].

**Figure 2:**
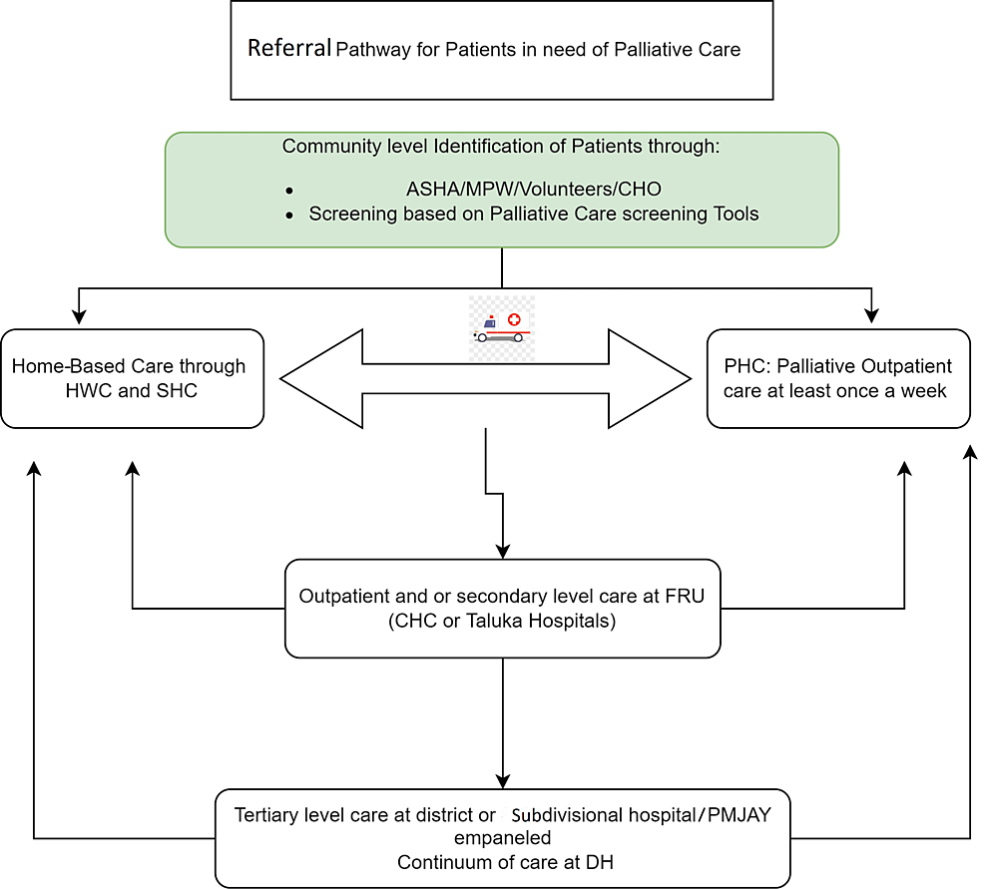
Algorithm for providing palliative treatment ASHA: Accredited social health activist; MPW: Multipurpose health worker; CHO: Community health officer; HWC: Health and Wellness Center; PHC: Primary health centers; FRU: First referral unit; CHC: Community health centers; PMJAY: Pradhan Mantri Jan Arogya Yojana; DH: District hospital. Image credit: The authors of the current study.

The algorithm to provide palliative treatment at different levels in India is shown in Figure 2 [23]. The algorithm can summarize it as the patient being identified through various levels by Accredited Social Health Activist (ASHA), Multipurpose Health Worker (MPW), Volunteers, Community Health Officer (CHO), and screening of people. These patients can choose to receive palliative care at home or at the Primary Health Center's (PHC) outpatient care facility. Responsible medical personnel can refer these patients to Taluka hospital for secondary level care or to a subdivisional hospital for tertiary level care [23].

Steps Involved in Palliative Care

**Figure 3:**
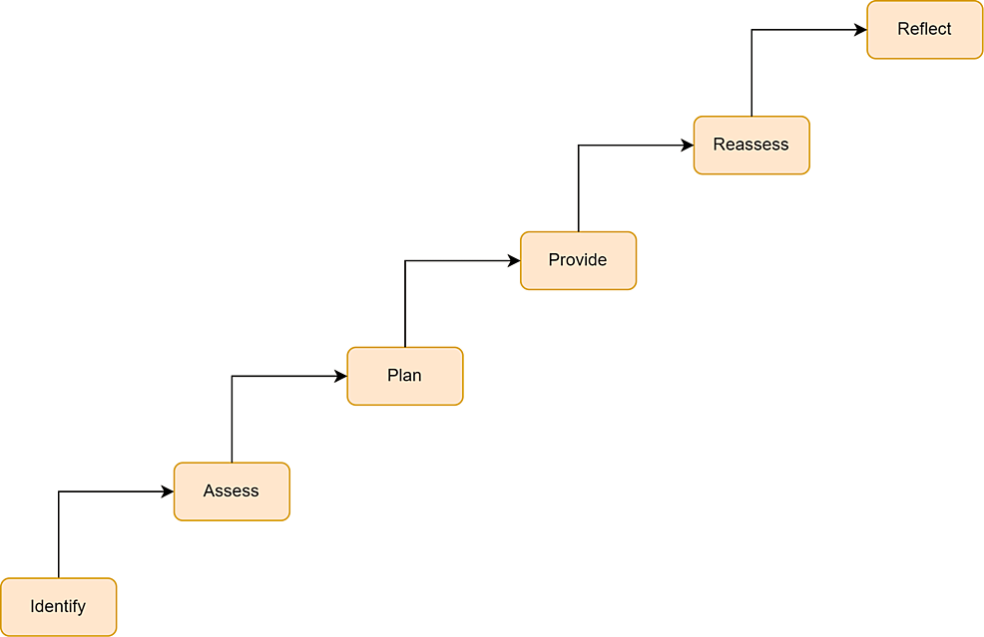
Steps involved in palliative care Image credit: The authors of the current study.

To provide palliative care, one must first identify the patient and then assess the patient's condition through proper history and appropriate investigations. After that, the care which is supposed to be provided to the patient should be planned, and facilities should provide care. Then, medical personnel should reassess the patient to know the care's acceptance and effect. The reflected findings should be recorded and documented for future use. This process is shown diagrammatically in Figure 3 [22].

Key hurdles to the advancement of palliative care

As an outcome of the discussion above, the primary elements that contribute to the need for healthcare system development in the respected field include a lack of policies and political support, insufficient funding for development, and inadequate training for health professionals. The general public lacks a solid understanding of the actual relevance of palliative care [24,25]. Although the population and service demand are growing, the capacity for training is not. By 2030, there must be an adequate medical workforce for hospice and palliative treatment services, which would require an increased number of hospice and palliative medicine fellowship program holders (HPM fellowship degrees) from the current annual average of 325 to somewhere between 500 and 600 [26]. Misaligned (fee-for-service) payment schemes partly create a lack of organizational capability and an absence of monitoring, accreditation criteria, and regulatory obligations to assure access and quality.

Evidence reveals that consumers prefer to employ complementary therapies, including naturopathy, Ayurveda, Siddha medicine, and Unani (herbal treatment). These alternative therapies are preferred because of the unavailability of opioids, lack of knowledge about alternatives, budgetary constraints, a lack of nearby resources, variable service quality, poor continuity of care, among other factors [27], and lack of sufficient data to support the safe and efficient conduct of available services. There are also cultural obstacles such as extra-human factors that have hampered the development of facilities that provide palliative care. Hospice, palliative, and end-of-life care are now associated with death and dying. Psychologically, most people avoid death because they are terrified of it. People frequently believe that it is harmful even to ponder dying soon. This claim is unsupported by any data, and research indicates that palliative care may even improve survival rates [28,29].

Discussion

To achieve the goal of developing palliative care, inexpensive and high-quality pain management should be made available and easily accessible to the poor as a vital component of health care at all levels and community needs.

Palliative Care Calls for Three Levels of Expertise

All medical personnel, including doctors, nurses, mental health specialists, clergy, volunteers, and therapists, should receive basic palliative care training [30]. Intermediate training should be provided for people who consistently assist patients with life-threatening conditions [30]. Training in specialist palliative care should be provided for individuals who will teach palliative care and conduct research as well as for patients with more sophisticated symptom management demands [30]. The programs aim to use the existing healthcare system to deliver palliative care to patients to increase the output of the healthcare system and healthcare personnel and anticipate national programs under a single roof. The solutions described would offer significant funding for expanding crucial health programs for non-communicable chronic diseases, including malignancy, chronic respiratory disease, diabetes, multidrug-resistant tuberculosis, AIDS, and initiatives aimed at older populations. The collaboration between the finance and health ministries will also ensure that access to opioids for medicinal and research reasons is permitted under national laws and regulations [31].

The responsible and compatible authorities should take care of the regulatory issues related to expanding the accessibility of morphine, as stated in the program. National and international organizations that provide palliative care will work together to ensure the program's execution. The main recommendations are providing funds to create a governmental palliative treatment house and palliative care facilities at the neighborhood hospital [31]. Education, advocacy, and discussion are the only ways to bring about a social awakening. The Indian Society of Critical Care Medicine (ISCCM) and the Indian Association of Palliative care (IAPC) must serve as catalysts in this process by offering guidance and leadership. To promote an informed, ethical discussion, they must interact with patient groups, lawmakers, the press, opinion leaders, and the general public [32].

Home-care services

**Figure 4:**
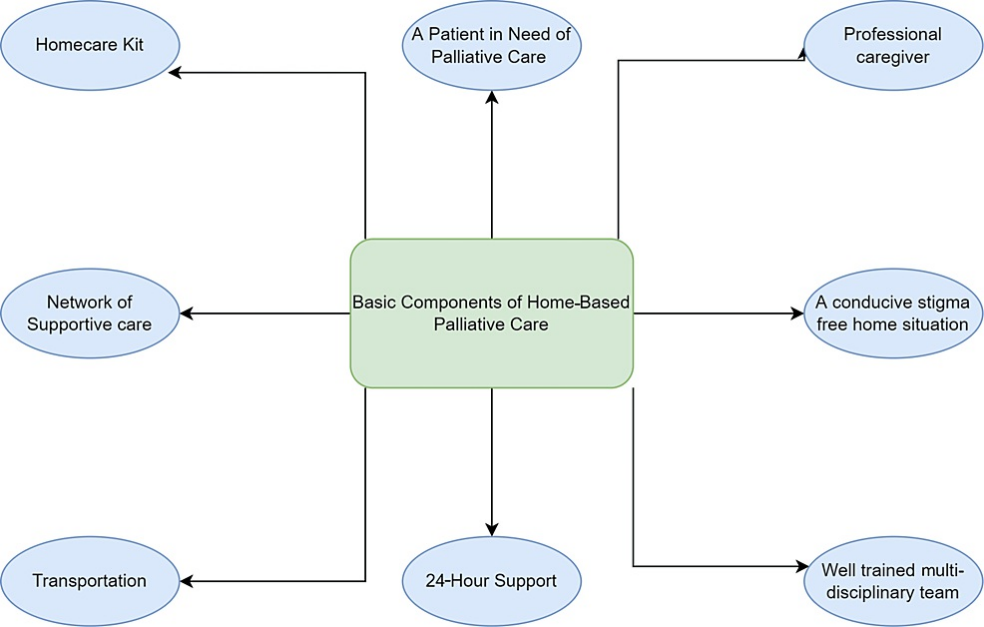
Requirements for home-care services Image credit: The authors of the current study.

One of the most significant hurdles in providing economically affordable palliative treatment to the people of this enormous subcontinent may be partially solved by establishing the services on the level of an individual home in rural as well as urban settings. The Neighborhood Network in Palliative Care (NNPC) project was created due to the home-care program's success [18]. Inspired by the critical features of home-care services mentioned in Figure 4, NNPC was an outstanding achievement. Although it could draw some clear conclusions regarding the cost-effectiveness of other therapies, the cost-effectiveness of the home-based palliative treatment center is proven [33]. In order to give complete long-term care and palliative care to the underprivileged in developing nations, the NNPC aims to create a sustainable "community-driven" service. Neighborhood volunteers are taught in this program to recognize issues faced by locals with degenerative illnesses and successfully address them with a community of knowledgeable specialists. NNPC aims to give local communities with the resources they need to care for chronically ill and dying individuals. It is influenced by the Alma-Ata Declaration's primary healthcare proposal by the World Health Organization [34].

## Conclusions

There are too many obstacles to developing and achieving the gold standard of palliative care, including variables like concentration of people, illiteracy, topographical density, stringent prescription drug laws, essential skill building, limited national policy, and institutional disinterest in palliative care. India as a country shows an interesting mentality, which says palliative care is meant for the economically higher class of the population. India has to understand that everyone has the right to live their lives to the fullest, possibly happily and without any regret.

However, all these hurdles in developing and redesigning the basic structure and number of palliative care in India have been improving daily for the last two decades. States like Kerala are role models for developing home-based services. Experimental trials of a newer model of facilities providing palliative care have been conducted in Mumbai and Pune in Maharashtra. These step-by-step improvements will lead to developing a solid foundation for the emerging better future of palliative treatment-providing facilities in India.
